# Beyond the Gender Binarism: Neural Correlates of Trans Men in a Functional Connectivity–Resting-State fMRI Pilot Study

**DOI:** 10.3390/jcm13195856

**Published:** 2024-09-30

**Authors:** Giuseppe Maniaci, Giorgio Collura, Caterina La Cascia, Tommaso Piccoli, Eleonora Bongiorno, Ilaria Barresi, Maurizio Marrale, Cesare Gagliardo, Alessandra Giammanco, Valeria Blandino, Crocettarachele Sartorio, Stefano Radellini, Laura Ferraro, Francesca Toia, Giovanni Zabbia, Giulia Bivona, Massimo Midiri, Marcello Ciaccio, Daniele La Barbera, Adriana Cordova, Diego Quattrone

**Affiliations:** 1 Section of Psychiatry, Department of Biomedicine, Neuroscience, and Advanced Diagnostics, University of Palermo, 90127 Palermo, Italy; erika.lacascia@unipa.it (C.L.C.); eleonorabongiorno@gmail.com (E.B.); ilariabarresi94@gmail.com (I.B.); alessandra.giammanco@unica.it (A.G.); rachele.sartorio@unipa.it (C.S.); laura.ferraro@unipa.it (L.F.); daniele.labarbera@unipa.it (D.L.B.); diego.quattrone@unipa.it (D.Q.); 2 Department of Physics and Chemistry, University of Palermo, 90127 Palermo, Italy; giorgio.collura01@unipa.it (G.C.); maurizio.marrale@unipa.it (M.M.); 3 National Institute of Nuclear Physics, Section of Catania, 95125 Catania, Italy; 4 Section of Neurology, Department of Biomedicine, Neuroscience and Advanced Diagnostics, University of Palermo, 90127 Palermo, Italy; tommaso.piccoli@unipa.it (T.P.); valeriablandino0@gmail.com (V.B.); 5 Section of Radiological Sciences, Department of Biomedicine, Neuroscience and Advanced Diagnostics, University of Palermo, 90127 Palermo, Italy; cesare.gagliardo@unipa.it (C.G.); massimo.midiri@unipa.it (M.M.); 6 Department of Medical Sciences and Public Health, University of Cagliari, 09040 Cagliari, Italy; 7 Section of Endocrinology, Department of Health Promotion, Maternal-Infantile, Internal and Specialist Medicine of Excellence “G. d’Alessandro” (PROMISE), University of Palermo, 90127 Palermo, Italy; radellinistefano@gmail.com; 8 Division of Plastic and Reconstructive Surgery, Department of Surgical, Oncological and Oral Sciences, University of Palermo, 90127 Palermo, Italy; francesca.toia@unipa.it (F.T.); zabbia.giovanni@libero.it (G.Z.); adriana.cordova@unipa.it (A.C.); 9 Department of Biomedicine, Neurosciences and Advanced Diagnostics, Institute of Clinical Biochemistry, Clinical Molecular Medicine and Clinical Laboratory Medicine, University of Palermo, 90127 Palermo, Italy; giulia.bivona@unipa.it (G.B.); marcello.ciaccio@unipa.it (M.C.); 10 Social, Genetics and Developmental Psychiatry Centre, Institute of Psychiatry, Psychology and Neuroscience, King’s College London, London SE5 8AF, UK

**Keywords:** gender dysphoria, fMRI, functional connectivity, gender incongruence, resting state, trans

## Abstract

**Introduction**: Several studies have investigated the specific neural correlates of trans people, highlighting mixed results. This study aimed to compare the presence of specific functional connectivity and differences in cognitive profile and hormone levels in trans men diagnosed with gender dysphoria (GD), and a homogeneous group of cisgender men and cisgender women. **Methods**: A total of 42 participants (19 trans men, 11 cisgender men, and 12 cisgender women) underwent a resting state fMRI and were measured for blood levels of testosterone, estradiol, and progesterone. A neuropsychological battery evaluated executive functions, attention, visual-perceptual ability, verbal fluency, manual preference, and general intelligence. **Results**: Trans men showed weaker functional connectivity in the precentral gyrus, subcallosal cortex, paracingulate gyrus, temporal pole, and cingulate gyrus than cisgender men (*p* < 0.01). Trans men performed worse than cisgender men in verbal and visuospatial working memory but similarly to cisgender women (*p* < 0.05). In trans men, functional connectivity of the precentral gyrus correlated positively with testosterone (*r* = 0.459, *p* = 0.064) and negatively with estradiol (*r* = −0.654, *p* = 0.004) and progesterone blood levels (*r* = −0.475, *p* = 0.054). The cluster involving the subcallosal cortex showed a positive correlation with testosterone (r = 0.718, *p* = 0.001), and a negative correlation with estradiol (r = −0.602, *p* = 0.011). The functional connectivity from a cluster involving the paracingulate gyrus showed a positive correlation with testosterone (*r* = 0.592, *p* = 0.012). **Conclusions**: This study highlights the importance of overpassing the binary model by underlining the presence of neural pathways that could represent the peculiarity of the neural profile of people with GD.

## 1. Introduction

The term “trans” is an umbrella for people whose gender identity does not align with the birth-assigned sex [1]. Some trans individuals may experience gender dysphoria (GD), a strong and persistent cross-gender identification and a long-standing discomfort with their birth-assigned sex [2].

Both biological [3,4] and psychosocial [5] factors are involved in the etiology of GD. Structural neuroimaging studies highlighted neuroanatomical similarities between trans and cis individuals sharing the same birth-assigned sex, such as the total brain volume [6], grey matter volume [7], regional cortical thickness [8], and cortical morphometry [9]; however, other authors found no differences between these two groups (e.g., Simon and colleagues) [10]. Furthermore, trans women had a cerebral activation pattern similar to cisgender women under erotic stimuli [11], whereas trans men showed a masculinization of brain structures associated with visuospatial functions [12,13]. Biological sex, gender and sexual orientation influence cognitive abilities [14] but few studies have investigated individual differences in cognitive functioning [15]. Some studies investigated the hormonal role in cognitive functions between trans individuals, especially executive functions, working-memory and attentional domains [16]. In a longitudinal study involving trans men, Gómez-Gil et al. [17] found an activating effect for androgens on visual memory, a domain that generally tends to favor cis men. Moreover, a recent study showed that trans men scored lower on episodic memory than cisgender women but scored equal to cisgender men [18]. However, some studies have concluded that assuming hormone therapy results in no change in verbal or visual spatial performance among trans people [19,20].

Interestingly, trans individuals showed peculiarities in structural and functional patterns implicated in body perception [10], such as the insula [6], the cingulate cortex, precuneus cortex, mesial prefrontal cortex, the left angular gyrus, and the superior parietal cortex [21,22,23,24,25,26]. Clemens and colleagues [27] examined the effect of hormonal treatment on resting state functional connectivity (rs-FC) of subjects with GD, finding a stronger FC in the thalamus of hormone-naïve trans women compared with treated ones and cis women; however, after receiving hormone therapy, their rs-FC shifted more toward their aspired gender. Santarnecchi et al. [28] replicated this finding on a single hormone-naive trans man. In this regard, hormone therapy seems to render the brain structural connectivity more similar to individuals who share the same gender identity.

These changes were observed in the right superior longitudinal fasciculus, the right corticospinal tract [29], the hypothalamus [30], areas involved in body perception [25,31], and neural networks involved in visuospatial functions [13], verbal fluency [32], cognitive processes related to empathy and mentalizing [33].

Currently, the scientific community is not unanimous in supporting the need to move beyond the binary gender model. This lack of consensus is partly due to the limited evidence in the literature highlighting the neurological, psychological, and endocrinological distinctiveness of individuals with gender dysphoria (GD).

In particular, as far as we know, there is a lack of studies evaluating simultaneously the rs-fMRI, cognitive profile and hormone levels in a sample of trans-men with GD compared to cisgender men and women [12,13]. In our opinion, the contextual evaluation of these three factors allows for the identification of any specific characteristics of trans individuals, ensuring a better framework for diagnosis and clinical management. Therefore, in this study we sought to compare the group of trans individuals with that of cis women and cis men through an integrated assessment, including fMRI, hormones levels and neuropsychological tests.

Specifically, our objectives were to: (a) assess rs-FC in a sample of outpatients with GD using an independent component analysis (ICA) approach; (b) investigate the cognitive profile of trans men; (c) examine the relationship between the re-FC measures, blood levels of testosterone, estradiol, progesterone, and the cognitive profile in trans participants.

## 2. Materials and Methods

### 2.1. Study Procedure and Participants

For this case-control study, between February 2019 and December 2020, 25 trans men (13 hormone-naïve), 14 cisgender women, and 14 cisgender men consented to participate in the study. Trans men were recruited from the Department of Plastic Surgery of the A.O.U.P. “P. Giaccone”, as they were seeking care to begin their gender affirmation process, whereas, to facilitate snowball sampling, cis participants were enrolled through word-of-mouth recruitment. Specifically, cis volunteers were recruited by responding to an advertisement posted in the outpatient clinic, and by inviting friends of the trans participants.

Participants underwent, on the same day, a blood sample collection to evaluate the hormonal profile of testosterone, estradiol and progesterone and a neuropsychological assessment and, the next day, an rs-fMRI session.

Trans individuals fulfilled the diagnostic criteria for GD according to the DSM-5.

Both trans and cis participants met the following inclusion criteria: aged between 18 and 55; having capacity to provide written informed consent. Exclusion criteria were: having an intelligence quotient (IQ) lower than 65; any severe psychiatric and neurological disorders; currently receiving pharmacological treatment for a mental health condition; having first-degree relatives with a history of psychotic disorders; having any cerebral lesions. All participants were right-handed, except for one left-handed individual in each group. All participants were asked about their sexual orientation and gender identity. Specifically, sexual orientation was assessed by asking to participants: “Do you consider yourself to be: heterosexual or straight, gay or lesbian, bisexual, not sure/questioning, something else”.

Concerning hormone therapy, trans men had been receiving testosterone enanthate 250 mg every 3–4 weeks titrated, until blood testosterone values were in the normal range for the cis male population of the same age.

All participants provided their written informed consent, and all study procedures were administered with due regard to participants’ privacy.

This study was granted ethical approval from the ethical review board of the Policlinico “P. Giaccone” of Palermo, Italy (Verb. 11/2019). All procedures involving human participants were performed in accordance with the ethical standards of the institutional and national research committee and with the 1964 Declaration of Helsinki and its later amendments.

### 2.2. Measures

#### 2.2.1. Screening Measures

The Edinburgh Handedness Inventory identified manual preference [34,35]. A short version [36] of the Italian version of WAIS-R [37], including Digit Symbol, Arithmetic, Information, and Block Design, assessed IQ.

#### 2.2.2. Neuropsychological Assessment

All participants underwent a neuropsychological evaluation of four cognitive domains in line with previous studies [17,38]: executive function, attention, visual-perceptual ability, and verbal fluency. We used the Digit and Corsi Span Forward (FW) and Backward (BW) versions [39] to investigate working-memory performances and a component of executive function. The cut-off scores for the Italian population are 4.26 for the Digit Span forward task, 2.65 for the Digit Span backward task, 3.4 for Corsi Span forward, and 3.08 for Corsi Span backward.

The Trail Making Test (TMT) [40] was used to evaluate attentional skills in a visual research task and the subject’s ability to quickly switch from a numerical stimulus to an alphabetic one. The cut-off score was <94 for Part A, <283 for Part B, and <187 for Part (B–A) score.

The Stroop Color-Word Test-short version [41] examined selective attention by the subject’s ability to inhibit conflicting/interfering responses. The cut-off scores were 36.92 for the time interference effect (I.T.) and 4.24 for the error interference effect (I.E.), respectively.

The Poppelreuter–Ghent overlapping figures Test (PGT) [42] was used as visual recognition task of meaningful and meaningless two-dimensional line-drawings of shape, investigated visual-perceptual ability. The cut-off scores were 28.06 for common objects (MF), 23.25 for abstract figures (ML), and 51.54 for total score (Tot PGT).

The Verbal Fluency Test (FAS) [43], considered as an executive function index, evaluated the access to the phonemic lexical store. The cut-off score was 17.35.

#### 2.2.3. MRI Acquisition

All participants underwent brain MRI scans using a 1.5 Tesla MRI unit (SignaHDxt; GE Medical System, Milwaukee, WI, USA) at the Radiology Section of the Department of Biomedicine, Neuroscience and Advanced Diagnostics of the University of Palermo. We used an eight-channel brain-phased array coil. To reduce head motion during image acquisition, foam pads were placed around the participants’ heads and within the head coil. An MRI technician with over fifteen years of experience explained and gave suggestions to each patient on how to keep the head still throughout the examination. Structural images were obtained through a T1-weighted sagittal three-dimensional (3D) 1.2 mm thick Fast Spoiled GRadient-echo (FSPGR) prepped inversion recovery pulse sequence (acquisition matrix 256 × 256; slice thickness 1.2 mm; TR 12.4 ms; TE 5 ms; IT 450 ms; FA 20; parallel imaging method: Array coil Spatial Sensitivity Encoding, ASSET). Rs-fMRI data were acquired using a two-dimensional (2D) axial T2*-weighted gradient-echo Echo-Planar (EP) pulse sequence parallel to the anterior commissure-posterior commissure (AC-PC) line covering the entire brain (acquisition matrix 64 × 64; 33 slices; slice thickness 3 mm; gap 1 mm; TR 3000 ms; TE 60 ms; FA 90); the first five scans were discarded to allow T1 saturation to reach equilibrium; a ten-minute (200 volumes) fMRI scan was performed on each participant. All subjects were instructed to remain motionless during the scan, to quietly rest in the scanner with their eyes open, and to avoid thinking about anything in particular. Scan parameters were consistent for all imaging sessions. Prior to the FC analyses, a neuroradiologist with twenty years of experience evaluated the images to exclude incidental findings and artifacts (not only motion-related) that could affect the results of the analyses. No patient was excluded due to the detection of incidental findings or artifacts following visual analysis by the expert neuroradiologist.

### 2.3. Resting-State fMRI Data

The rs-fMRI data preprocessing was performed following the pipeline adopted in literature [44,45], using FSL version 5.0.9 (www.fmrib.ox.ac.uk/fsl accessed on 10 April 2016). Various components of the FSL software were used in the process. The steps involved in preprocessing resting-state data included: motion correction with the MCFLIRT tool, slice-timing adjustment via Fourier-space phase-shifting, removal of non-brain elements using BET, spatial smoothing with a Gaussian kernel of 6.0 mm FWHM, normalizing the mean intensity of each volume over time, high-pass temporal filtering with a sigma of 100.0 s, and pre-whitening, and global spatial smoothing with a Gaussian kernel of 6 mm FWHM. Subsequently, the processed images were aligned with the corresponding high-resolution echo planar images, which were co-registered with T1-weighted images and then matched to the MNI-152 standard space image of 2 mm isotropic resolution through a non-linear registration with 12 degrees of freedom using FLIRT, and further refined with FNIRT warping. Motion distortion was evaluated in individual time-series to determine the inclusion or exclusion of fMRI data in the final group analysis. The threshold of estimated translations for motion artifacts detection was set to 0.5 mm (MCFLIRT tool). In this work, excessive motion was considered if the estimated translation was larger than 0.5 mm along any axis. No patient was excluded due to supra-threshold motion. Movement parameters calculated by MCFLIRT were modelled as nuisance covariates.

Consistently with the exploratory nature of this study, we used a data-driven approach. Probabilistic independent component analysis (PICA) was performed through the FSL’s MELODIC toolbox. As recommended for resting state data analysis, the multi-session temporal concatenated ICA (Concat-ICA) approach was chosen. The groups considered were compared in pairs with the aim of looking for significantly different regions. Then, the subjects of each couple of groups considered were concatenated for the ICA. According to the literature [45,46], 40 independent components (IC) maps were extracted. The inference on estimated maps was accomplished through a mixture model performing variance normalization, thresholding IC maps and checking the local false-discovery rate at *p* < 0.5. The different component maps are tested voxel-wise for statistically significant differences between the groups using FSL dual regression. In particular, FSL randomized non-parametric permutation testing with 10,000 permutations, applying a threshold-free cluster enhancement technique (TFCE) for more sensitive detection of cluster-like structures, while still preserving the voxelized nature of the images. Correction for multiple comparisons across space was applied, assuming a global significance level of less than 0.05, using permutation testing and TFCE.

### 2.4. Statistical Analyses

Group-based differences in terms of age, IQ, neuropsychological tests, and hormone levels were examined using a one-way analysis of variance (ANOVA). Group-based differences with respect to sexual orientation, marital status, level of education, employment status and handedness were examined using χ² tests. A Pearson correlation analysis was performed to examine the relationship between the ICA, hormone levels and neuropsychological tests. A post hoc analysis using a Bonferroni correction for multiple testing was performed.

All analyses assumed an alpha risk of 5%. All statistical analyses were conducted using IBM SPSS Statistics for Windows, version 22.0 (Armonk, NY, USA: IBM Corp.).

## 3. Results

### 3.1. Sociodemographic Variables

The final sample included 42 subjects: 19 trans men (8 hormone-naïve), 11 cis men, and 12 cis women. No participants had any physical condition that could affect cognitive performance (for a review, see Tragantzopoulou and Giannouli [47]). One participant was excluded because of a cerebellar lesion detected during the rs-fMRI session, one because of the presence of a borderline personality disorder, and nine because of motion artefacts. Trans and cis participants were comparable in terms of age F(2,38) = 0.613, *p* = 0.547; sexual orientation χ² (4) = 7.129, *p* = 0.129; marital status χ² (6) = 7.199, *p* = 0.303; level of education χ² (6) = 11.700, *p* = 0.069; employment status χ² (2) = 3.632, *p* = 0.163; handedness χ² (2) = 0.084, *p* = 0.959; and IQ F(2,35) = 1.615, *p* = 0.213.

As expected, we observed differences in testosterone F(2,37) = 10.325, *p* ≤ 0.00001, and in estradiol levels F(2,37) = 3.448, *p* = 0.042, and to a lesser extent in progesterone levels F(2,37) = 3.028, *p* = 0.061 (Table 1). Post hoc Bonferroni correction confirmed higher testosterone in trans men (M = 5.95, SD = 5.18, *p* = 0.001) and cis men (M = 6.27, SD = 2.48) than in cis women (M = 0.36, SD = 0.09, *p* = 0.001).

### 3.2. IC Analysis and Dual Regression

Dual regression analysis showed statistically different FC in IC7, IC11, and IC20.

**Figure 1:**
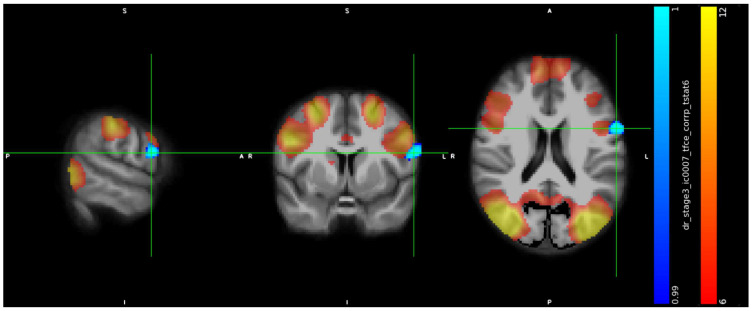
Increased RS-FC in cis men compared to trans men in the left Precentral Gyrus, Inferior Frontal Gyrus, pars opercularis (peak at x, y, z: 75, 66, 46, *p* < 0.01, cluster size = 169 voxels).

IC7 included a cluster in the precentral gyrus (peak at x, y, z: 75, 66, 46, cluster size = 169 voxels) (Figure 1) and showed in trans men weaker connectivity than in cisgender men (*p* < 0.01).

**Figure 2:**
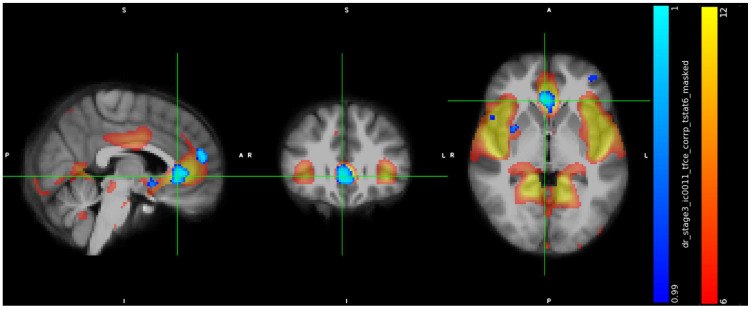
Increased RS-FC in cis men compared to trans men in the Subcallosal Cortex and Cingulate Gyrus, anterior division (peak at x, y, z: 43, 78, 35, *p* < 0.01, cluster size = 664 voxels).

**Figure 3:**
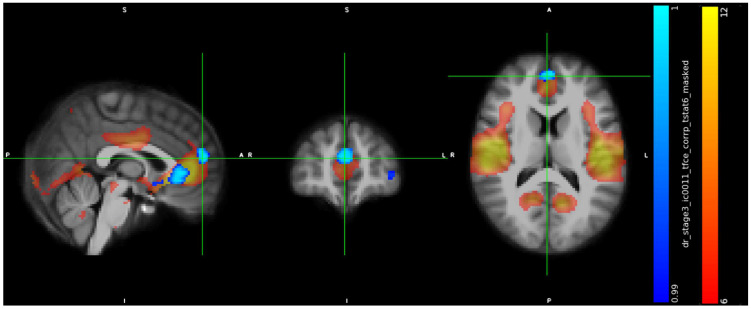
Increased RS-FC in cis men compared to trans men in the Paracingulate Gyrus, Frontal Pole and Superior Frontal Gyrus (peak at x, y, z: 44, 89, 43, *p* = 0.01, cluster size = 170 voxels).

**Figure 4:**
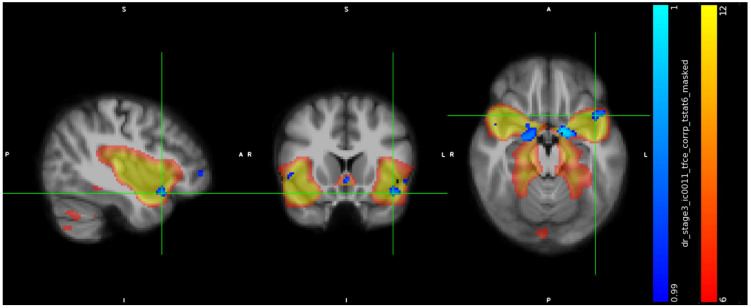
Increased RS–FC in cis men compared to trans men in the left Temporal Pole, Frontal Orbital Cortex, Insular Cortex (peak at x, y, z: 66, 71, 27, *p* < 0.01, cluster size = 142 voxels).

IC11 included a cluster in the subcallosal cortex (peak at x, y, z: 43, 78, 35, cluster size = 664 voxels) (Figure 2), a cluster in paracingulate gyrus (peak at x, y, z: 44, 89, 43, cluster size = 170 voxels) (Figure 3), a cluster in temporal pole (peak at x, y, z: 66, 71, 27, cluster size = 142 voxels) (Figure 4), and showed in trans men a weaker FC than cisgender males (*p* < 0.01).

**Figure 5:**
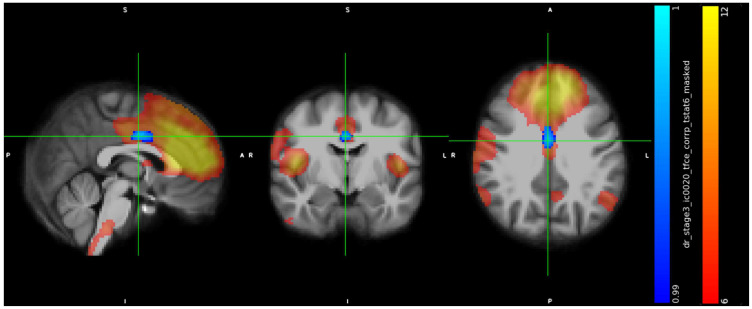
Increased RS-FC in cis men compared to trans men in the Cingulate Gyrus, anterior division and Cingulate Gyrus, posterior division (peak at x, y, z: 44, 60, 53, *p* < 0.01, cluster size = 157 voxels).

IC20 included a cluster in the cingulate gyrus (peak at x, y, z: 44, 60, 53, cluster size: 157 voxels) (Figure 5) and showed in trans men a weaker FC than cis men (*p* < 0.01).

### 3.3. Neuropsychological Data

The ANOVA analyses showed differences in Digit Span Backward F(2,30) = 6.856, *p* = 0.004, Corsi Span Forward F(2,30) = 5.693, *p* = 0.008, and Corsi Span Backward F(2,30) = 5.028, *p* = 0.013, all higher in cis men than cis women and trans men. Visuospatial, attention, executive, and language functions were similar among the three groups.

Post hoc analysis of Digit Span BW data showed that trans men (*p* = 0.022) and cis women (*p* = 0.005) performed worse than cis men. We found similar results with Digit Span FW (*p* = 0.055). Equally, in Corsi Span FW, trans men (*p* = 0.029) and cis women (*p* = 0.014) performed worse than cis men. However, in Corsi Span BW, only trans men performed worse than cis men (*p* = 0.015). Corsi Span BW between cis men and cis women gave similar results (Table 2). Results show that the cognitive profile of trans men is more similar to that of cis women than cis men, even when controlling for hormonal therapy.

### 3.4. ICA, Neuropsychological Data, and Hormones

In trans men, a negative correlation was identified between the precentral gyrus activation (ICA 7) and performance on the TMT (*r* = −0.597, *p* = 0.04).

Moreover, we observed in trans men a negative correlation between activation in a cluster involving the subcallosal cortex (ICA 11) and performance on the Stroop IT (*r* = −0.626, *p* = 0.03), as well as a negative correlation between activation in the paracingulate gyrus (ICA 11) and the performance on the Stroop IT (*r* = −0.591, *p* = 0.043). Furthermore, a cluster involving the cingulate gyrus in ICA 20 was positively correlated with performance on the FAS verbal fluency (*r* = 0.713, *p* = 0.009).

In cisgender women, a negative correlation was observed between the precentral gyrus (ICA 7) and the performance on the Corsi BW test (*r* = −0.697, *p* = 0.025); furthermore, a cluster involving the cingulate gyrus (ICA 20), was positively correlated with Stroop IE (*r* = 0.805, *p* = 0.005).

In trans men, the activation of the precentral gyrus (ICA 7) was negatively correlated with estradiol (*r* = −0.654, *p* = 0.004) blood levels. Moreover, a cluster involving the subcallosal cortex (ICA 11) was positively correlated with testosterone (*r* = 0.718, *p* = 0.001), and negatively with estradiol (*r* = −0.602, *p* = 0.011). Finally, a positive correlation between the cluster involving the paracingulate gyrus (IC 11) and testosterone was found (*r* = 0.592, *p* = 0.012).

No correlations were found within the cisgender men group.

## 4. Discussion

Although the brain is a mosaic of male and female features [48], in recent years, numerous neuroscience studies have investigated the neurobiological correlates of GD finding heterogeneous results [49]. An interesting theory implies that there might exist brain phenotypes for trans people [50]. In this regard, Smith et al. [49] suggested that the brain of trans individuals does not seem to be entirely feminized or masculinized. Several brain areas are well known for their peculiar FC in trans people. For instance, Savic and Arver [51] found in trans women a brain pattern similar to people sharing the same birth-assigned sex, while according to Simon and colleagues [10] the precentral gyrus of both trans women and trans men does not differ from controls sharing the same gender identity.

In literature, there is a partial disagreement regarding the question of whether trans individuals more closely resemble those with whom they share a gender identity or those with whom they share a birth-assigned sex [23,49]. In our study, we used rs-fMRI to investigate the specific neural correlates of a group of trans men with GD compared with a group of cisgender men and women homogeneous for age, level of education, handedness, sexual orientation, marital, and employment status.

The relationship between brain connectivity and cognitive function in the context of GD is still a matter of debate and results from early papers are not conclusive, probably due to the different cognitive tasks that authors used or the different complexity of the same tasks [52,53,54,55]. Moreover, other features may contribute to the conflicting nature of these results, such as the activities performed during the development of brain connectivity [56,57], different activities of daily life, such as physical activity [58], as well as environmental, biological, and neurobiological factors [59].

We found weaker FC in trans than in cis men in the cingulate gyrus, a brain area involved in body perception [60], and in structures engaged in the Default Mode Network (DMN) such as the paracingulate gyrus and the subcallosal cortex, which is engaged in cognitive and emotional processes [61]. These areas could be influenced by hormone therapy and, especially within the subcallosal cortex, trans women showed a deactivation during estradiol treatment [62]). Furthermore, in trans individuals, the posterior cingulate cortex, which seems to be more similar to people sharing their gender identity [10], is one of the main structures engaged in the emotional significance of body-self alignment of trans individuals and related to their motivation toward hormone and/or gender-affirming surgery [63].

In addition, we found weaker FC in the right precentral gyrus in trans than in cis men. This result is not consistent with Simon et al. [10], who found that the precentral gyrus of both trans women and trans men do not differ from controls sharing the same gender identity.

We also found that trans men have weaker FC than cisgender men in the temporal pole. This finding is consistent with Clemens et al. [27], who reported diminished FC in the temporal lobe of hormone-naïve trans women compared to cis men. These results are consistent with the literature suggesting that the temporal pole plays an essential role within the auditory network and expresses gender differences in morphometric connectivity [64].

In line with our study, Feusner et al. [24] suggested a different pattern in trans people compared to cisgender ones on FC within the DMN and the visual network, both engaged in the self- and own-body awareness and perception. Moreover, the activation of the posterior cingulate gyrus and the lateral occipital cortices, the precuneus and the angular gyrus was also associated with higher levels of body uneasiness during a gender face discrimination task [53]. Therefore, our results are consistent with the literature suggesting that connectivity in areas involved in body perception may be specific neural correlates of GD. Considering what has been discussed and based on our results, it seems that the brain areas involved in the mental representation of body image and the alignment between this representation and the actual body image are anatomically and functionally distinct in individuals with GD, and are influenced by hormones. Therefore, within a clinical-therapeutic framework, it is important to recognize that these individuals have brain characteristics that differ from those both of cis men and women. Furthermore, hormone therapy plays a critical role in driving functional changes and shaping the body image representation in trans individuals.

The second aim of this study was to examine variation in the cognitive profile across the three groups. Current evidence supports that gender variation in the cognitive performance attributable to gender is small, particularly in developed countries [65]. However, cis men generally perform better than cis women in visuospatial abilities [66], while cis women perform better than cis men in verbal and memory tasks [13]. These differences may be mainly attributed to the different spatial competence [66] and to the circulating hormonal variation [67].

We reported a better performance in cisgender men compared to cisgender women and trans men in tasks assessing verbal and visuospatial working-memory. We reported no variation in other cognitive domains, including verbal fluency, attention, and visuospatial perception. Contrary to the assumption that cognitive function in trans individuals aligns more closely with their gender identity counterpart [10,68], our findings suggest a similar cognitive profile between trans men and cisgender women. The contrast between our data on cognitive profiles and the existing literature may suggest that a range of biological and environmental factors contribute to the alignment of trans individuals with cisgender individuals who share the same gender identity. Consequently, while it remains unclear what specifically drives these discrepancies, future research could explore variables, such as hormone therapy or prenatal hormonal state, to shed light on the potential underlying mechanisms.

Finally, our third aim was to examine the associations between FC measures, blood levels of testosterone, estradiol and progesterone, and cognitive profile in the trans participants. Notably, FC in the precentral gyrus among trans men was positively correlated with testosterone levels and negatively correlated with estradiol and progesterone levels. Mueller et al. [52] proposed that intrinsic brain activity correlates with fluctuations in sex steroid and reported that, in trans men, testosterone and androstenedione were positively associated with increased low-frequency fluctuation in the precentral gyrus, a component of the frontoparietal network. Clemens et al. [27] identified variation in this network between hormone-naïve and hormone-treated trans women, further highlighting the effects of hormone therapy. Furthermore, a cluster within the subcallosal cortex was positively correlated with testosterone and negatively correlated with estradiol. This result is consistent with Schneider et al. [62], who reported reduced activity within the subcallosal cortex following 60 days of estradiol treatment, thus emphasizing the role of sex hormones in modulating FC in GD. Several studies have documented significant changes on FC of different areas of the brain in trans women after gender-affirming hormone therapy; in a recent study, Reed and colleagues [69] found that the right insula (seed region) showed increased connectivity with the posterior cingulum, left middle frontal gyrus and left angular gyrus after hormone therapy in the trans women group when compared to cis men. Moreover, in trans men, a positive correlation was observed between testosterone and FC in the paracingulate gyrus; this structure increases its connectivity after hormone treatment [31]. This data suggests a significant sensitivity of various brain areas to hormone therapy in individuals with GD. This finding is particularly important within the context of treatment pathways aimed at improving the quality of life for trans individuals. The changes on FC in trans men are less studied and the potential effect of the hormone therapy on this topic need to be well explained. Therefore, future studies should aim to investigate how hormone levels affect neural activity and assess the broader implications of these changes on the well-being of trans people.

To the best of our knowledge, this is the first study to assess the rs-FC and cognitive profile of a sample of trans individuals with GD. The main limitation of this study pertains to the small sample size, which included only outpatients who desire to undergo gender affirming surgery. Moreover, the sample size was not previously estimated. While snowball sampling is an effective method to enroll trans participants, this may not fully capture the variability within the broader trans population. Snowball sampling can result in participants with similar characteristics being overrepresented. To enhance sample diversity, we recruited participants who came from different demographic and social backgrounds. Therefore, while the findings offer valuable insights, they should be interpreted within the context of these sampling constraints. We did not evaluate the timing of blood draws in both hormone-naive transmen and cisgender women relative to the day of the menstrual cycle, which could introduce variability in hormone levels. However, since we did not account for this timing in any of the subjects, we do not know if significant differences are present. Lastly, we grouped both hormone-naïve and treated transmen together for data analysis. However, considering the specific characteristics of the population under investigation, the recruitment of a large cohort of trans individuals who fulfill all the inclusion and exclusion criteria remains challenging.

Furthermore, the present study recruited a group of cisgender individuals homogenous in terms of age, sexual orientation, marital status, level of education, employment status, handedness, and IQ. Future investigation should include a larger sample and evaluate the timing of blood draws relative to the day of the menstrual cycle; moreover, they could explore the potential modifications in neural pathways and their FC both preceding and subsequent to gender-affirming surgical interventions.

## 5. Conclusions

In our study, we found variation between trans and cis men in brain regions implicated in body image perception, including the precentral gyrus, subcallosal cortex, paracingulate gyrus, temporal pole, and cingulate gyrus. Additionally, differences were observed in verbal and visuospatial working-memory, with cisgender men showing a better performance compared to both cisgender women and trans men. Reflecting on the need to transcend binary classification of gender and to embrace the concept of gender identity as a spectrum (Bradford et al., 2018), we can assume that a relevant unifying feature among trans individuals with GD may be the profound discomfort and uneasiness associated with their body perception. In this regard, it is noteworthy that both hormonal and surgical interventions are known to mitigate the discomfort related to body image [70], which is reflected in modifications in the brain structures involved in the body image networks.

## Figures and Tables

**Table 1:** Socio-demographic information of the sample.

Factor	Trans Men (N = 18) *M* (*SD*)	Cis Men (N = 11) *M* (*SD*)	Cis Women (N = 12) *M (SD)*	ANOVA	*p* Value
**Age**	26.22 (10.28)	23.54 (2.97)	23.91 (2.46)	0.613	0.547
**IQ**	91.50 (17.41)	91.90 (8.40)	99.54 (9.34)	1.388	0.263
**Testosterone**	5.95 (5.18)	6.27 (2.48)	0.36 (0.09)	10.325	0.000
**Estradiol**	80.11 (74.76)	29.11 (9.01)	95.35 (73.25)	3.448	0.042
**Progesterone**	1.59 (2.53)	0.39 (0.14)	4.57 (7.19)	3.028	0.061
	**Trans Men (N = 16)** ***Frequency* (*%*)**	**Cis Men (N = 11)** ***Frequency* (*%*)**	**Cis Women (N = 12)** ***Frequency* (*%*)**	**χ^2^ of Pearson**	***p*** **Value**
**Marital Status**					
**Single**	7 (46.7%)	5 (45.5%)	2 (16.7%)		
**In a stable relationship**	6 (40%)	6 (54.5%)	10 (83.3%)	7.199	0.303
**Married**	1 (6.7%)	-	-		
**Separated**	1 (6.7%)	-	-		
**Sexual orientation**					
**Homosexual**	-	1 (9.1%)	-		
**Heterosexual**	13 (81.2%)	10 (90.9%)	12 (100%)	7.129	0.129
**Bisexual**	3 (18.8%)	-	-		
					
**Level of education**					
**Middle school diploma**	6 (37.5%)	-	1 (8.3%)		
**High school diploma**	10 (62.5%)	11 (100%)	9 (75%)	11.700	0.069
**Bachelor’s degree**	-	-	1 (8.3%)		
**Master’s degree**	-	-	1 (8.3%)		
					
**Employment status**					
**Student**	7 (46.7%)	9 (81.8%)	12 (100%)		
**Unemployed**	4 (26.7%)	-	-	11.904	0.018
**Employed**	4 (26.7%)	2 (18.2%)	-		
					
**Handedness**					
**Left-handed**	1 (6.2%)	1 (9.1%)	1 (8.3%)	0.084	0.959
**Right-handed**	15 (96.8%)	10 (90.9%)	11 (91.7%)		

**Table 2:** Cognitive profiles and hormone levels comparison between the groups.

Factor	Trans Men (N = 17) *M (SD)*	Cis Men (N = 11) *M (SD)*	Cis Women (N = 12) *M (SD)*	df	ANOVA (*p* Value)	Partial η²	Post hoc Bonferroni
**PGT_MF**	35.91 (0.28)	36.00 (0.00)	35.9 (0.31)	*2, 30*	*F* = 0.513 (0.604)	0.033	-
**PGT_ML**	33.75 (1.76)	34.81(0.40)	34.6 (0.96)	2, 30	*F* = 2.489 (0.100)	0.142	-
**PGT_TOT**	69.66 (1.82)	70.81 (0.40)	70.5 (0.84)	2, 30	*F* = 2.734 (0.08)	0.154	-
**PGT_CFA**	0.75 (0.62)	0.54 (0.52)	0.50 (0.97)	2, 30	*F* = 0.389 (0.681)	0.025	-
**Trail Making Test (A)**	50.00 (14.75)	37.18 (11.0)	39.8 (13.89)	2, 30	*F* = 2.971 (0.066)	0.165	-
**Trail Making Test (B)**	91.33 (38.77)	73.45 (27.28)	73.2 (22.62)	2, 30	*F* = 1.298 (0.288)	0.080	-
**Trail Making Test (B-A)**	41.33 (33.39)	36.27 (23.12)	33.4 (20.95)	2, 30	*F* = 0.250 (0.781)	0.016	-
**Verbal Fluency Test**	37.91 (8.03)	38.90 (10.16)	45.8 (11.75)	2, 30	*F* = 1.949 (0.160)	0.115	-
**Stroop Test (IT)**	8.08 (8.20)	9.25 (7.08)	9.5 (6.38)	2, 30	*F* = 0.121 (0.886)	0.008	-
**Stroop Test (IE)**	0.33 (0.91)	0.09 (0.30)	0.20 (0.42)	2, 30	*F* = 0.436 (0.651)	0.028	-
**Digit Span—Forward**	6.25 (0.75)	6.63 (1.12)	5.6 (0.96)	2, 30	*F* = 3.151 (0.057)	0.174	-
**Digit Span—Backward**	4.75 (1.13)	6.09 (1.22)	4.40 (0.96)	2, 30	*F* = 6.856 (0.004)	0.314	CisM > transM, cisM > cisW
**Corsi Span—Forward**	5.66 (0.88)	6.72 (0.90)	5.5 (0.97)	2, 30	*F* = 5.693 (0.008)	0.275	CisM > tansM, cisM > cisW
**Corsi Span—Backward**	4.66 (0.65)	5.63 (0.80)	5.4 (0.84)	2, 30	*F* = 5.028 (0.013)	0.251	CisM > transM, cisM > cisW
**Testosterone**	5.95 (5.18)	6.27 (2.48)	0.36 (0.09)	2, 30	*F* = 10.325 (0.000)	-	CisM > cisW, transM > cisW
**Progesterone**	1.59 (2.53)	0.39 (0.14)	4.57 (7.19)	2, 30	*F* = 3.028 (0.061)	-	-
**Estradiol**	80.11 (74.76)	29.11 (9.01)	95.35 (73.25)	2, 30	*F* = 3.448 (0.042)	-	-

Note. df: degrees of freedom; CI [LL, UL]: Confidence intervals [lower-limit, upper-limit]; PGT_MF: Poppelreuter-Ghent’s overlapping figures test, Misidentification Figures; PGT_ML: Poppelreuter-Ghent’s overlapping figures test, Mislocalizations; PGT_TOT: Poppelreuter-Ghent’s overlapping figures test, Total; PGT_CFA: Poppelreuter-Ghent’s overlapping figures test, Correct Figure Assignments; IT: Interference Time; IE: Interference Effect. Confidence intervals for: PGT_MF: Trans men versus cis men CI 95% [−0.3438, 1.771]; trans men versus cis women CI 95% [−0.2505, 0.2838]. PGT_ML: Trans men versus cis men CI 95% [−2.3542, 0.2179]; trans men versus cis women CI 95% [−2.1692, 0.4692]. PGT_TOT: Trans men versus cis men CI 95% [−2.4450, 0.1420]; trans men versus cis women CI 95% [−2.1602, 0.4935]. PGT_CFA: Trans men versus cis men CI 95% [−0.5557, 0.9648]; trans men versus cis women CI 95% [−0.5299, 1.0299]. TMT_A: Trans men versus cis men CI 95% [−1.3080, 26.9443]; trans men versus cis women CI 95% [−4.2900, 24.6900]. TMT_B: Trans men versus cis men CI 95% [−14.7955, 50.5530]; trans men versus cis women CI 95% [−15.3824, 51.6491]. TMT_B_A: Trans men versus cis men CI 95% [−23.3172, 33.4384]; trans men versus cis women CI 95% [−21.1753, 37.0420]. FAS: Trans men versus cis men CI 95% [−11.5550, 9.5702]; trans men versus cis women CI 95% [−18.7180, 2.9513]. STROOP_IT: Trans men versus cis men CI 95% [−8.9257, 6.5833]; trans men versus cis women CI 95% [−9.3709, 6.5376]. STROOP_IE: Trans men versus cis men CI 95% [−0.4179, 0.9028]; trans men versus cis women CI 95% [−0.5440, 0.8107]. Digit Span Forward: Trans men versus cis men CI 95% [−1.3942, 0.6214]; trans men versus cis women CI 95% [−0.3837, 1.6837]. Digit Span Backward: Trans men versus cis men CI 95% [−2.5253, −0.1566]; trans men versus cis women CI 95% [−0.8648, 1.5648]. Corsi Forward: Trans men versus cis men CI 95% [−2.0336, −0.0876]; trans men versus cis women CI 95% [−0.8314, 1.1647]. Corsi Backward: Trans men versus cis men CI 95% [−1.7807, −0.1587]; trans men versus cis women CI 95% [−1.5652, 0.0986].

## Data Availability

The raw data supporting the conclusions of this article will be made available by the authors on request.
